# Exploring the Use of Social Media for Activism by Mexican Nongovernmental Organizations Using Posts From the 16 Days of Activism Against Gender-Based Violence Campaign: Thematic Content Analysis

**DOI:** 10.2196/67368

**Published:** 2025-04-17

**Authors:** Marian Marian, Ramona L Pérez, Elizabeth Reed, Samantha Hurst, Rebecka Lundgren, Amanda C McClain, Kathryn M Barker

**Affiliations:** 1 University of California San Diego La Jolla, CA United States; 2 San Diego State University San Diego, CA United States

**Keywords:** gender-based violence, Mexico, hashtag activism, feminist social activism, hashtag feminism, Twitter, X, nongovernmental organization, social media

## Abstract

**Background:**

In the past decade, *hashtag feminism* has emerged in Mexico as a prevalent strategy to build social movements against gender-based violence (GBV). For example, during the global “16 Days of Activism Against GBV” campaign held between November 25 and December 10 each year, Mexico-based nongovernmental organizations (NGOs) turn to X (formerly known as Twitter) to share messages. Despite this prevalence, there is limited research on the type of information shared by these NGO activists on social media and the public’s engagement with these messages.

**Objective:**

This study aims to explore the themes covered by Mexican NGOs on X and examine what types of messages related to GBV potentially resonated more with the public.

**Methods:**

We collated and reviewed posts (commonly known as tweets) published in Spanish on the platform X by Mexico-based NGOs between November 25 and December 10 of 2020, 2021, and 2022, a period when digital interactions increased during the COVID-19 pandemic. We then extracted posts using the following 4 hashtags: #16días, #16DiasdeActivismo, or #16DíasdeActivismo; #25N or #25Noviembre; #DiaNaranja or #DíaNaranja; and #PintaElMundoDeNaranja. We subsequently assessed the number of likes each post had and retained the top 200 posts from each year with the highest number of likes. We used the iterative content analysis process and the inductive 6-step qualitative thematic analysis method in NVivo software to code and analyze the final 600 posts.

**Results:**

Five themes emerged from the 16 Days of Activism Against GBV campaigns, covering both knowledge-sharing and activism-generating messages as follows: (1) activism and how to be an activist, (2) types of GBV most commonly highlighted in posts, (3) changing public discourse surrounding GBV, (4) GBV as a violation of human rights, and (5) the COVID-19 pandemic’s impact on GBV. Most of the messages on these posts exclusively mentioned women and younger girls, while a few included adolescents. Gaps in the representation of vulnerable populations were also found.

**Conclusions:**

The posts from this campaign that were highly liked by the public reflect some of the most significant societal issues currently present in the country. Our results could help guide further GBV campaigns. Still, further research related to hashtag feminism by Mexico-based NGOs on GBV is needed to understand the population that NGOs reach and how the messages shared on these campaigns translate into activism on online and offline social media platforms.

## Introduction

### Background

Violence against women and girls, also commonly referred to as gender-based violence (GBV), is one of the world’s most persistent forms of human rights violations and a major problem in the public health and clinical health arenas [1,2]. Specifically, GBV continues to be a serious problem in Mexico, as 70% of the women aged ≥15 years have experienced at least one incidence of GBV throughout their lives [3]. GBV takes many forms, with the most prevalent forms in Mexican society being psychological and sexual violence [3]. Disappearances and femicide, which is the intentional murder of a girl or woman because of her gender, are also common forms of violence in Mexico, with approximately 25% of women, adolescent girls, and young girls experiencing these forms of violence [4,5]. The COVID-19 pandemic increased the risk of violence against women, adolescents, and girls; women in North America, Central America, and South America were disproportionately affected by GBV during the pandemic [6]. Particularly in Mexico, domestic violence increased by more than 15% between 2020 and 2021 [7]. In 2020, the number of emergency calls related to violence against women increased by >30%, and the requests for support due to GBV increased by 40% [8,9]. Forms of structural GBV, such as violence at the social and economic levels, were also impacted during the COVID-19 pandemic in Mexico, for example, by the continuous increase in unpaid domestic and care work [10]. In addition, digital violence, which involves gender-based acts of violence through communication technology or digital media, such as image-based abuse, cyberstalking, blackmailing, and online harassment, increased in visibility in Mexico in the years surrounding the limitations of social interactions [10-13].

In Mexico, government institutions provide some forms of social support in the fight against the various forms of GBV, although important gaps in coverage are addressed by nongovernmental organizations (NGOs). Specifically, NGOs provide health care, psychosocial support, and legal aid for women, adolescents, and girls experiencing this type of violence [14]. As GBV and gender inequality became more visible during the COVID-19 pandemic, feminist movements, such as hashtag activism, feminist social activism, and hashtag feminism, in which NGOs actively collaborate, also became more visible [15]. Hashtag activism can facilitate social change, policy formation, and the provision of resources for the public [16]. Feminist social activism, popular since the late 2000s and present in Mexico since 2011, involves feminist individuals and organizations using social media for online civic engagement to denounce GBV and related aspects (such as sexism and gender discrimination); initiate social movements; and connect to share experiences, support, and resources, to name a few [17].

Online engagement to denounce GBV and other forms of gender-inequitable practices includes the initiation of social movements by connecting individuals to share their experiences, provide support, and offer sources of support. Hashtag feminism is a form of activism that combines hashtag activism with feminist social activism using specific hashtags across different digital platforms to call for action by sharing information and stories, connecting people, and organizing and mobilizing events against gender inequities [18-20]. NGOs have played a significant role in catalyzing hashtag feminist movements on GBV worldwide and in Mexico by engaging stakeholders and the general public through dialogue and community-building practices, mainly through Facebook and X (formerly known as Twitter; X Corp) [21-29]. An example of this is the 16 Days of Activism Against GBV, an annual international campaign led by civil society that runs from November 25, the International Day for the Elimination of Violence Against Women, to December 10, Human Rights Day [30,31]. The intention of this global campaign is to call for the prevention and elimination of violence against girls, adolescents, and women in all its different forms, including child marriage, sexual harassment, and intimate partner violence, to name a few [31,32].

Hashtag feminist movements stand to make a great impact in Mexican society, as social media use is widespread. In 2021, 89.2% of the internet users in Mexico connected to the internet daily, and 89.8% used it to access social media [33]. As of January 2022, Mexico ranked ninth in the world for most X users, with 13.9 million users [34]. Similar to what happened in other countries, digital interactions increased during the COVID-19 pandemic in Mexico, and the use of social media to promote strategies against violence, such as support groups, helplines, and screening for violence, became more popular [35-37]. Despite the prevalent use of X in the world and in Mexico, research is limited in regard to hashtag activism and hashtag feminism through X on GBV, specifically on interpersonal and sexual violence [22,38-40]. To our knowledge, only a few publications exist on feminist social activism in Mexico that are specific to GBV at the local level [17,41]. Furthermore, the X content from campaigns that address the umbrella of different types of GBV by Mexico-based NGOs has not previously been explored. Understanding the public’s engagement on the GBV hashtag feminist campaigns is critical, as this can inform future GBV campaigns and provide valuable information to NGOs, governments, and researchers for tailored online and offline GBV prevention and intervention strategies.

### This Study

To fill this gap, this research aimed to describe the range of GBV themes that Mexican NGOs posted on X during the annual 16 Days of Activism Against GBV campaign during the COVID-19 pandemic (from 2020 to 2022) and elucidate what types of messages related to GBV the public engaged with the most across and between the 3 different years. It is important to specify that while in Mexico the more specific term “violence against women” is commonly used, as it is the term described in the General Law on Women’s Access to a Life Free from Violence first published in 2007 [3], this research followed the term GBV, as it is an umbrella term commonly used interchangeably [42] with violence against women and is the term used in the global campaign that is the focus of our investigation. We sought to describe the GBV themes raised by Mexico-based NGOs on X during these global campaigns and, based on the public’s engagement, how the main content themes of posts (commonly known as tweets) compare between the 3 different years. Our findings can provide information on how these campaigns and the public’s engagement could modify the agenda against GBV at different societal levels that was weakened during the COVID-19 pandemic [43] by contributing information on the topics that are generating the attention of the public, providing voice to certain communities, and adapting these results into current or future political or legal initiatives.

## Methods

### Study Design and Data Collection

Our research methodology followed a thematic content analysis of posts posted by Mexico-based NGOs during the annual 16 Days of Activism Against GBV campaign from 2020 to 2022. The following four hashtags chosen for this analysis were based on informal exploration on X of relevant hashtags used by Mexico-based NGOs during the campaign in 2020, 2021, and 2022: (1) the overall name of the campaign and its variations, namely #16días, #16DiasdeActivismo, or #16DíasdeActivismo (#16daysofactivism); (2) the first day of the campaign and the International Day for the Elimination of Violence Against Women (November 25), namely #25N or #25deNoviembre; (3) Orange Day, which occurs on the 25th of each month to create awareness and prevent violence toward girls and women, namely #DiaNaranja and #DíaNaranja (#OrangeDay); and (4) a related but broader hashtag, namely #PintaElMundoDeNaranja (#PaintTheWorldOrange). Given that the hashtag #10D, referring to December 10, the last day of the campaign and Human Rights Day, is also used annually in Argentina for democratic campaigns, we excluded it from analysis as a strategy to ensure data focused on the GBV campaign.

During data collection, the advanced search option from X was used to filter data based on the following study inclusion criteria: (1) only original content (no reposts or retweets); (2) included one of the hashtags used by Mexico-based NGOs during the campaign that were selected for this analysis (#16días, #16DiasdeActivismo, #16DíasdeActivismo, #25N, #25deNoviembre, #DiaNaranja, #DíaNaranja, or #PintaElMundoDeNaranja); (3) published in Spanish; (4) posted by a public X user with “non-governmental and nonprofit organization” as the professional category in order to only collect posts from local, state, national, or global NGOs; (5) the location of the X user marked as Mexico or a city or state in it; and (6) posted between November 25 and December 10 of 2020, 2021, and 2022, to align with the dates the campaign occurred. We used the Zeeschuimer and the 4CAT research tools to capture and download data from X. Zeeschuimer is a browser extension that collects data from social media sites, such as Instagram, TikTok, and X, while 4CAT is a research tool that can be connected to Zeeschuimer to store and download the data collected by the browser extension [44].

### Data Cleaning

Posts posted between November 25 and December 10 of 2020, 2021, and 2022 were downloaded from 4CAT to a Microsoft Excel spreadsheet in August 2023. Posts that included videos (<1 min) and informational graphics were also included in the analysis. A total of 1914 posts (n=662, 34.59% for 2020; n=654, 34.17% for 2021; and n=598, 31.24% for 2022) were collected for the hashtags and the selected time period. To avoid duplicates, identical posts published during the same campaign year were collapsed and considered as one individual post. The collected posts were reviewed to ensure they met the inclusion criteria (only posts in Spanish, X user being an NGO, and location of the X user in Mexico).

We used the number of likes received by a post as the metric to determine the public’s interest in and engagement with a post [45]. The frequency of likes has been previously used in the literature, including studies on GBV and feminist activism, to measure the public’s engagement [21,39,46-50]. Reposts, which have been considered another form of public engagement in which the public contributes and creates content through reposting or forwarding a post [22], were not included in the analysis in order to focus on an original and representative set of posts posted by Mexico-based NGOs that participated in the 16 Days of Activism Against GBV campaigns.

We limited the analysis to the top 200 posts per year with the most likes (N=600 total posts) to assess the messages that generated the most engagement from the public. This analytic decision was based on previously published literature that has demonstrated that at least 500 posts are sufficient to identify themes and understand how individuals engage in health behaviors on X [51,52]. In the event of identical posts from different NGOs, we collapsed the number of likes.

### Data Coding and Analysis

After obtaining and cleaning the data, the final list of 600 posts was uploaded into NVivo (version 14; Lumivero) for coding and analysis in the original language. We retained the original language of the posts to more accurately capture the cultural connotations and local meaning of the messages [53]. We completed an iterative content analysis process and a 6-step inductive thematic content analysis to identify and categorize the content of the posts into themes and explore socially produced and reproduced experiences [21,22,54]. These systematic approaches to interpreting qualitative research data have been used by other researchers when conducting thematic content analysis of social media data, including X hashtags in GBV campaigns [38,55-59]. The thematic process involved familiarization with the data, selection of keywords, coding, theme development, interpretation of themes, and development of a conceptual model [55]. An iterative inductive content analysis approach [60] was followed for the creation of the codebook by the first author and 2 research assistants (Yxchel Tejeda and Julia Godinez), as code categorization and subcategorization were extracted from the posts. Both research assistants were bilingual and were trained in thematic content analysis. Regular debriefing meetings between the primary author and the 2 research assistants took place during the coding and analysis process to refine the working codebook, discuss findings and emergent themes, resolve any coding discrepancies, and address any potential questions. The research team used NVivo (version 14) for coding and analysis. After the initial analysis, data were translated from Spanish to English by the first author (MM), who is bilingual.

### Ethical Considerations

This study used publicly available, deidentified posts and did not directly involve human participants. Per the US Department of Health and Human Services' regulations for the protection of human subjects in research (45 CFR 46), approval from a research ethics committee was therefore not required or obtained. However, we adhered to ethical guidelines for handling and analyzing publicly available data, ensuring user anonymity and data privacy throughout the research process, including findings reported in this study.

## Results

### Overview

The 600 posts included in the final sample came from 61 different Mexico-based organizations. The number of likes for each post ranged from 9 to 2095. As shown in Table 1, the hashtag using the campaign’s name (16 Days of Activism Against GBV) was the most frequently used hashtag in all 3 campaign years.

**Table 1:** Frequency of hashtags mentioned on most liked posts per campaign year by Mexico-based nongovernmental organizations.

Hashtag	2020, n (%)	2021, n (%)	2022, n (%)	Total, n (%)
#25N or #25Noviembre (n=216)	56 (25.9)	58 (26.9)	102 (47.2)	216 (100)
#DiaNaranja or #DíaNaranja (n=283)	113 (39.9)	111 (39.2)	59 (20.8)	283 (100)
#16días, #16DiasdeActivismo, or #16DíasdeActivismo (n=528)	167 (31.6)	204 (38.6)	157 (29.7)	528 (100)
#PintaElMundoDeNaranja (n=76)	49 (64.5)	22 (28.9)	5 (6.6)	76 (100)

The following five themes emerged across the 3 campaign years: (1) activism and how to be an activist, (2) types of GBV most commonly highlighted in posts, (3) changing public discourse surrounding GBV, (4) GBV as a violation of human rights, and (5) the impact of the COVID-19 pandemic on GBV. Within some of these themes, we also identified several cross-cutting subthemes, as described subsequently.

### Theme 1: Activism and How to Be an Activist

The most common theme among the highly liked posts covered descriptions of what the 16 Days of Activism Against GBV campaign is, along with what activism is and how to be an activist (Table 2). Many of these posts were invitations to join the campaign or be activists. Other posts stressed that activism to support the rights of women and girls does not stop when the campaign stops but should be practiced on a regular basis. Invitations, live updates, and links to different types of events, such as marches, podcasts, and webinars organized during the campaigns, were also shared in these posts.

**Table 2:** Representative quotes from theme 1 (activism and how to be an activist).

Subthemes	Examples (posts translated into English)	Examples (posts in original language)
Description of the campaign	“During these #16Días and all year long, stand in solidarity with women’s rights activists and support feminist movements to resist the rollback of women’s and girls’ rights. #Únete #25N” [2022 campaign] “What are the #16Días de activismo? It is a call to action and a reminder that violence against women and girls is the most widespread human rights violation around the world. #DíaNaranja Take action to end violence against women and girls.” [2022 campaign]	“Durante estos #16Días y todo el año, solidarízate con las activistas de los derechos de las mujeres y apoya a los movimientos feministas para resistir el retroceso de los derechos de las mujeres y las niñas. #Únete #25N” [Campaña 2022] “¿Qué son los #16Días de activismo? Es un llamado a la acción y un recordatorio de que la violencia contra las mujeres y las niñas es la violación de derechos humanos más extendida en todo el mundo. #DíaNaranja Actúa para poner fin a la violencia contra las mujeres y las niñas.” [Campaña 2022]
Campaign events	“Women musicians, visual artists, activists, journalists, photographers and filmmakers join their voices to create #25NMás16, a sound, visual and informative piece promoted by the Initiative #SpotlightMX 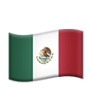 #DíaNaranja #Únete #16Días” [2020 campaign]	“Mujeres músicas, artistas visuales, activistas, periodistas, fotógrafas y cineastas unen sus voces para crear #25NMás16, una pieza sonora, visual e informativa impulsada por la Iniciativa #SpotlightMX 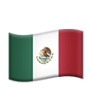 #DíaNaranja #Únete #16Días” [Campaña 2020]
Geographical examples	“#25N| This is a fight for all women 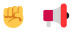 ‘Neither in Tijuana, nor in Chiapas, nor in this city, NOT ONE MORE MURDERED’ 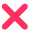 #NiUnaMenos #VivasNosQueremos” [2020 campaign]	#25N| Esta es una lucha de todas las mujeres 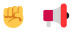 “Ni en Tijuana, ni en Chiapas, ni en esta ciudad, NI UNA ASESINADA MÁS” 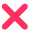 #NiUnaMenos #VivasNosQueremos” [Campaña 2020]

**Figure 1:**
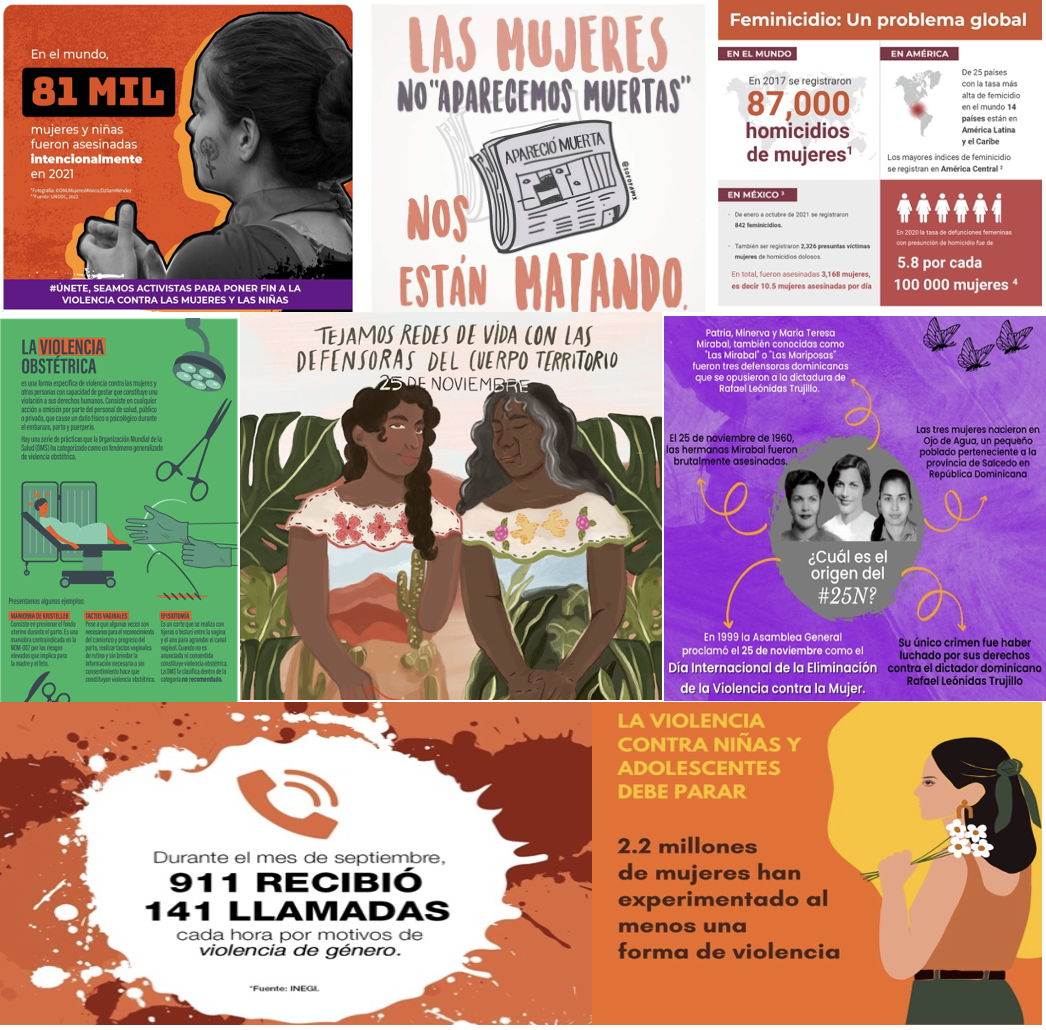
Examples of visuals used by Mexico-based nongovernmental organizations in the frequently liked X (formerly known as Twitter) posts for the 16 Days of Activism Against Gender-Based Violence campaigns in 2020, 2021, and 2022.

Different NGOs used different types of communication materials to explain the campaign’s purpose and why and how to be an activist, including songs, short videos, and celebrity involvement. Particularly in the 2021 campaign, posts included images and quotes related to the campaign from musicians, politicians, ambassadors, NGO directors and representatives, and leaders in industry and sport. Figure 1 shows examples of visuals used in highly liked posts for the 16 Days of Activism Against GBV campaigns in 2020, 2021, and 2022.

Posts’ messages on activism and how to be an activist were mainly generalized to the national level or provided data at a country level, although some included specific activities and examples of GBV cases from different municipalities, states, and, in 3 instances, other countries.

### Theme 2: Types of GBV Most Commonly Highlighted in Posts

Many posts consisted of information about a specific type of violence (Table 3). Sexual violence was the most common type of violence found in these posts, followed by messages related to trafficking, disappearances, and femicide. These types of posts included data on the number of femicides or women and girls who have disappeared in the country and advice on what needs to change to prevent trafficking. Specific examples of women who have experienced GBV, particularly sexual violence and torture, were also included. Violence at home and physical violence were other forms of GBV that were also commonly present in these posts. Posts about digital violence were present in the data as well, particularly in 2020, at the start of the COVID-19 pandemic.

**Table 3:** Representative quotes from theme 2 (types of gender-based violence most commonly highlighted in posts).

Subthemes	Examples (posts translated into English)	Examples (posts in original language)
Sexual violence	“Let us join forces against those who attempt to blur the boundaries of sexual consent, blame victims, and excuse perpetrators. During the #16Days of Activism, let’s say it loud and clear: NO IS NO. #Únete #DíaNaranja” [2020 campaign]	“Unamos nuestros esfuerzos contra quienes intenten desdibujar los límites del consentimiento sexual, culpar a las víctimas y excusar a los agresores. Durante los #16Días de Activismo, digámoslo alto y claro: NO ES NO. #Únete #DíaNaranja” [Campaña 2020]
Femicides and trafficking	“ 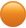 Mexico closes the year with the terrible number of 27 thousand missing girls and women. Within the framework of #16Días of Activism, it is essential to demand differentiated strategies with a gender perspective to find them all” [2022 campaign] “ 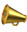 In Mexico, 11 femicides occur DAILY. Today #25N International Day for the Elimination of Violence against Women, we raise our voices for freedom, security, justice and sisterhood for all girls and women in Mexico. 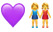 #NiUnaMas #VivasNosQueremos #DiaNaranja” [2020 campaign]	“ 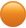 México cierra el año con la terrible cifra de 27 mil niñas y mujeres desaparecidas. En el marco de #16Días de activismo es fundamental exigir estrategias diferenciadas con perspectiva de género para encontrarlas a todas” [Campaña 2022] “ 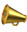 En México ocurren 11 feminicidios DIARIOS. Hoy #25N Día Internacional de la Eliminación de la Violencia contra la Mujer, alzamos la voz por la libertad, seguridad, justicia y sororidad para con todas las niñas y mujeres en México. 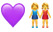 #NiUnaMas #VivasNosQueremos #DiaNaranja” [Campaña 2020]
Violence at home	“Women, girls and adolescents need to have peace of mind in their home and feel that their home is a safe and reliable space. Their home is NOT a space of #violence. Let’s make families and homes #EspaciosSeguros! #25N #PintaElMundoDeNaranja #16Días” [2021 campaign]	“Las mujeres, niñas y adolescentes necesitan tener tranquilidad en su hogar y sentir que su casa es un espacio seguro y confiable. Su hogar NO es un espacio de #violencia. ¡Hagamos de las familias y los hogares #EspaciosSeguros! #25N #PintaElMundoDeNaranja #16Días” [Campaña 2021]
Digital violence	“The increase in internet use during #COVID19 has made women and girls targets of online violence. In these #16Días of Activism, #pintaelmundodenaranja using your networks to raise awareness and promote solidarity. #DíaNaranja #Únete” [2020 campaign]	“El incremento del uso de internet durante #COVID19 ha convertido a mujeres y niñas en blanco de violencias en línea. En estos #16Días de Activismo, #pintaelmundodenaranja usando tus redes para concientizar y fomentar la solidaridad. #DíaNaranja #Únete” [Campaña 2020]
Physical violence	“ 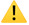 Did you know that 1 in 3 women in the world has suffered physical or sexual violence throughout their lives? Share this post and #Únete the #16días of activism to end this global pandemic. #25N” [2021 campaign]	“ 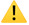 ¿Sabías que 1 de cada 3 mujeres en el mundo ha sufrido violencia física o sexual a lo largo de su vida? Comparte este post y #Únete a los #16días de activismo para acabar con esta pandemia mundial. #25N” [Campaña 2021]

Other forms of violence, such as the lack of access to sexual and reproductive resources, obstetric violence, vicarious violence, and precarious work, were present in posts, but they were not as common as sexual violence, femicide, and digital violence. Violence against women in the form of harassment in specific locations, such as school and at work, was also highlighted by the campaign but to a lesser extent.

### Theme 3: Changing Public Discourse Surrounding GBV

Posts in this theme centered on changing current narratives in Mexican society surrounding GBV that blame and silence survivors and normalize violence (Table 4). The posts provided examples of how to change the narrative, such as believing the victim, not excusing the perpetrator, and reporting any form of violence. The actors responsible for making these changes included both individuals and institutions. At the individual level, most posts were not targeted at a specific gender; however, some did aim toward male individuals and their role in eradicating GBV. Some highly liked posts were focused on female individuals, while other posts were specific to inviting both men and women to become activists and eradicate GBV. Throughout the 3 campaign years, the highly liked posts provided specific information about GBV toward women or women and younger girls. A few highly liked posts included adolescents in the messages, and, when included, these posts were about violence against younger girls and adolescents or against younger girls, adolescents, and women. At the institutional level, posts provided reasons for the importance of these groups in combating GBV, such as health and government personnel providing help to women after experiencing some form of GBV, while also providing examples of what these institutions can do, such as treating survivors of GBV with dignity and defending the rights of women, adolescents, and girls.

**Table 4:** Representative quotes from theme 3 (changing public discourse surrounding gender-based violence).

Subthemes	Examples (posts translated into English)	Examples (posts in original language)
Believe survivors	“Every time a woman talks about her experience of sexual violence and we don’t believe her, rape culture grows stronger. Every time you hear a survivor’s story: 1-Listen. 2-Believe. 3-Support. #DíaNaranja #16Días #Únete” [2020 campaign]	“Cada vez que una mujer habla de su experiencia de violencia sexual y no le creemos, la cultura de la violación se hace más fuerte. Cada vez que escuches la historia de una sobreviviente: 1-Escucha. 2-Cree. 3-Apoya. #DíaNaranja #16Días #Únete” [Campaña 2020]
End the silence	“If you face violence, don’t be silent, raise your voice. Call 911 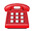 for help immediately and tell your trusted people. #16Días #DíaNaranja” [2021 campaign] “Report when you see: 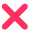 Abuse 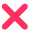 Harassment on the street 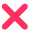 Sexist jokes 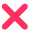 Unwanted behavior 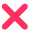 Inappropriate sexual comments Sexual harassment is never acceptable. #16Días #Únete #DíaNaranja” [2020 campaign]	“Si enfrentas violencia, no te calles, alza la voz. Pide ayuda inmediatamente al 911 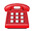 y cuéntalo a tus personas de confianza. #16Días #DíaNaranja” [Campaña 2021] “Denuncia cuando veas: 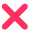 Abuso 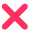 Acoso en la calle 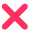 Bromas sexistas 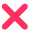 Comportamiento no deseado 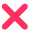 Comentarios sexuales inapropiados El acoso sexual nunca es aceptable. #16Días #Únete #DíaNaranja” [Campaña 2020]
Call out perpetrators’ actions	“Don’t be an accomplice to those who commit violence, don’t look the other way. Speak up, intervene and show your support for survivors during the #16Días, and every day. #Únete #DíaNaranja” [2020 campaign]	“No seas cómplice de quien ejerce violencia, no mires a otro lado. Habla, interviene y muestra tu apoyo a las sobrevivientes durante los #16Días, y todos los días. #Únete #DíaNaranja” [Campaña 2020]
Engage men	“As men we have to reflect, question ourselves, inform ourselves and act. #25N 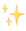 Let’s build relationships of respect, peace and equality! 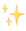 ” [2022 campaign]	“Como hombres nos toca reflexionar, cuestionarnos, informarnos y actuar. #25N 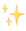 ¡Construyamos relaciones de respeto, paz e igualdad! 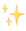 ” [Campaña 2022]
Focus on women and younger girls	“Eliminating violence against girls and adolescents is everyone’s task. Get informed and learn about different ways in which you can prevent gender violence. #16Días #DíaNaranja” [2021 campaign] “Violence will affect 1 in 3 girls and women throughout their lives. This must stop! #PintaElMundoDeNaranja and unite against violence.” [2020 campaign]	“Eliminar la violencia contra las niñas y las adolescentes es tarea de todas y todos. Infórmate y conoce diversas formas en que tú puedes prevenir la violencia de género. #16Días #DíaNaranja” [Campaña 2021] “La violencia afectará a 1 de cada 3 niñas y mujeres a lo largo de su vida. ¡Esto debe parar! #PintaElMundoDeNaranja y únete contra la violencia.” [Campaña 2020]
Responsibility of societal institutions	“People in leadership must implement prevention measures that address inequalities in power relations between genders, which are at the root of violence against women and girls #16Días #DíaNaranja” [2021 campaign] “Health providers can also defend the life and health of girls who have been forced to become mothers. Taking care of the health of survivors also involves having the ability to treat them humanely and with dignity. They are #GirlsNotMothers. #25N” [2021 campaign]	“Las personas en liderazgo, deben poner en marcha medidas de prevención que hagan frente a las desigualdades en las relaciones de poder entre los géneros, que se encuentran en la raíz de la violencia contra las mujeres y las niñas. #16Días #DíaNaranja” [Campaña 2021] “Las personas proveedoras de salud también pueden defender la vida y la salud de las niñas que han sido forzadas a ser madres. Cuidar la salud de las sobrevivientes también pasa por tener la capacidad de darles un trato humano y digno. Son #NiñasNoMadres. #25N” [Campaña 2021]

### Theme 4: GBV as a Violation of Human Rights

Posts related to GBV as a human rights concern rooted in gender inequalities and power imbalances were also common in the 3 campaign years (Table 5). These posts that stated the importance of challenging inequitable gender norms to promote equity and justice for girls and women were highly liked during the 3 campaign years. Particularly, for posts from 2020 related to justice and equity, many highly liked posts described real-life events of women in Mexico who had experienced sexual violence, wrongful convictions, and unjust incarcerations.

The focus on inequities did not stop at gender inequities. Posts also focused on violence toward specific vulnerable groups, such as people with disabilities, migrants, and sexual minority groups, and they were also found in the highly liked posts for the 3 campaign years, especially in 2021 and 2022. The two most common messages among posts related to vulnerable groups were (1) events to visualize the GBV that migrants experience in Mexico and (2) information on vulnerable populations being at a higher risk of experiencing GBV.

**Table 5:** Representative quotes from theme 4 (gender-based violence as a violation of human rights).

Subthemes	Examples (posts translated into English)	Examples (posts in original language)
Human rights violation	“ 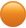 Violence against women is the most widespread violation of human rights in the world and the latest UN data show that the greatest threat to women and girls lies in the people in their immediate environment. #16Días #YaEsYa #DíaNaranja #Únete” [2022 campaign]	“ 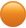 La violencia contras las mujeres es la violación más generalizada de los derechos humanos en el mundo y los últimos datos de ONU demuestran que la mayor amenaza para las mujeres y las niñas reside en las personas de su entorno más cercano. #16Días #YaEsYa #DíaNaranja #Únete” [Campaña 2022]
Origins in gender inequity	“The origin of violence is discrimination and gender inequality, as well as gender stereotypes and harmful masculinities still in force in our societies. #16Días #DíaNaranja #Únete” [2021 campaign]	“El origen de la violencia es la discriminación y la desigualdad de género, así como los estereotipos de género y las masculinidades nocivas aún vigentes en nuestras sociedades. #16Días #DíaNaranja #Únete” [Campaña 2021]
Violence toward vulnerable groups	“Inequality, poverty, ethnic origin, disability, immigration status, among others, increase the vulnerability of women and girls. Let’s end violence against women and girls NOW! #16Días #DíaNaranja” [2021 campaign]	“La desigualdad, la pobreza, el origen étnico, la discapacidad, el estatus #migratorio, entre otros, aumentan la vulnerabilidad de las mujeres y las niñas. ¡Pongamos fin a la violencia contra las mujeres y las niñas YA! #16Días #DíaNaranja” [Campaña 2021]

### Theme 5: The Impact of the COVID-19 Pandemic on GBV

Finally, a common theme among posts from 2020 (but not common in the 2021 and 2022 campaign posts) was the impact of the COVID-19 pandemic on GBV in Mexico. Posts in this theme highlighted the importance of the resources for GBV victims during this time and shared data or examples of how the pandemic increased GBV during the lockdown period in Mexico. Posts also cautioned that inaction could lead to setbacks in progress made toward gender equity. Table 6 presents selected quotations representing this theme.

**Table 6:** Representative quotes from theme 5 (the impact of the COVID-19 pandemic on gender-based violence).

Examples (posts translated into English)	Examples (posts in original language)
“We have stayed at home to avoid risks of contagion by #COVID19 but what happens if the risk is at home? Violence against women in the home has increased during the pandemic, but we can all help victims. #DíaNaranja #16Días #Únete” [2020 campaign]	“Nos hemos quedado en casa para evitar riesgos de contagio por #COVID19 pero ¿qué pasa si el riesgo está en casa? La violencia contra las mujeres en el hogar ha aumentado durante la pandemia, pero toda/os podemos ayudar a las víctimas. #DíaNaranja #16Días #Únete” [Campaña 2020]
“ 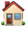 Shelters 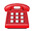 Helplines 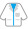 Advice Services for survivors are essential. Any type of support for victims of violence must be available to anyone who needs it, even during the #COVID19 pandemic. ^#^DíaNaranja #16Días #Únete” [2020 campaign]	“ 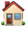 Refugios 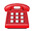 Líneas telefónicas de ayuda 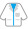 Asesoramiento Los servicios para las sobrevivientes son esenciales. Cualquier tipo de apoyo a las víctimas de violencia debe estar disponible para quien lo necesite, incluso durante la pandemia de #COVID19. #DíaNaranja #16Días #Únete” [Campaña 2020]
“The pandemic is being particularly painful for women. If no action is taken, the progress in gender equality made over the last 25 years will be lost. #16Días #DíaNaranja #Únete” [2020 campaign]	“La pandemia está siendo particularmente dolorosa para las mujeres. Si no se adoptan medidas, los avances en la igualdad de género logrados en los últimos 25 años se perderán. #16Días #DíaNaranja #Únete” [Campaña 2020]

## Discussion

### Principal Findings

Using data from 3 years of social media activism by Mexican NGOs on X, we found that messaging surrounding the 16 Days of Activism Against GBV centered on the following: (1) activism and how to be an activist, (2) types of GBV, (3) changing the public discourse surrounding GBV, (4) GBV as a human rights violation, and (5) the impact of the COVID-19 pandemic on GBV. The posts from this campaign that were highly liked by the public reflect some of the biggest societal issues currently present in Mexico. In total, 3 of the 5 main themes identified—the types of GBV, activism and how to be an activist, and changing public discourse surrounding GBV—were present across the 3 campaign years. The impact of the COVID-19 pandemic on GBV and GBV as a violation of human rights were the more prevalent themes in 2020 and 2021 and 2022, respectively. Subsequent research on future campaigns could help better understand how these themes evolve in Mexican society over time.

Activism and how to be an activist was the most common theme among the posts analyzed during the 16 Days of Activism against GBV campaigns from 2020 to 2022. The activism in this annual campaign educates the public on GBV through posts that show the information in different ways, such as videos, songs, data, infographics, and citing influential people, to name a few. Similar to what other studies have found related to digital activism, the posts from this campaign provide information that makes the problem of GBV visible, which helps destigmatize GBV in Mexico [61,62]. A mixed methods study published in 2018 analyzed whether GBV activism through Facebook creates sisterhood among followers and found that the sisterhood created on Facebook through the interaction and creation of groups and communities was translated into activism online and offline; this study noted that the lack of groups and communities on X does not allow this sisterhood to be created among X users [28]. However, we suggest that further analysis on X is needed to understand the potential of activism developing a sisterhood on this social media platform through reposts, replies, and other communication tools present on this platform.

Related to the different forms of GBV, sexual violence was the main form of violence highlighted in the analyzed posts. This resonates with the prevalence of sexual violence among the Mexican population, as this type of violence is the second most common form of GBV in Mexico [3]. Sexual violence prevalence among women aged ≥15 years increased from 41.3% in 2016 to 49.7% in 2021 [3]. Digital violence was another form of violence featured in the campaign posts. Digital violence soared in 2020 as a consequence of the COVID-19 pandemic [63]. This type of violence has continued to increase after the pandemic and affects girls and adolescent girls more than their male counterparts. Between 2021 and 2022, more than 33% of girls and adolescent girls in Mexico with access to a phone or internet experienced some form of digital violence, compared to 12% to 18% of Mexican boys and male adolescents [64]. As both forms of GBV continue to increase, the 16 Days of Activism against GBV campaign as well as other NGO- and non–NGO-based campaigns and prevention and intervention strategies should focus on sexual and digital violence in girls, adolescents, and women in Mexico.

We found that less commonly discussed forms of violence, such as economic violence, vicarious violence, lack of access to sexual and reproductive resources, and obstetric violence [65-69] were covered in the posts for these campaigns. Although other more commonly known forms of violence, such as sexual violence and intimate partner violence, were more commonly highlighted in these posts, having information on less commonly discussed forms of GBV within the campaign educated the public and provided tools to identify more types of violence. Indeed, although these forms of GBV are less commonly known by the public, it does not necessarily mean that these forms of violence are less prevalent. For example, Mexican national data have found that economic violence, which is not a well-known form of GBV in the country compared to physical, psychological, and sexual violence, is the most common form of GBV in the workplace [70].

A notable finding from theme 3, changing the narrative surrounding GBV, is that those called upon for change were not only individuals and the general public but also those in the public sector, including people in government leadership positions and health care providers. For the latter, the highly liked posts from the 3 campaign years acknowledged health care professionals as pillars of society for eradicating GBV in Mexico, one of the few countries with laws for the health care sector to address GBV at different prevention levels [71]. However, limited research has examined whether the Mexican health care sector is addressing GBV with their patients or working in other ways toward prevention. For example, a study completed among health care professionals in the states of Quintana Roo, Coahuila, and Mexico City found that while health care professionals were willing to address the issue of domestic violence with their patients, the care and attention needed for this specific type of violence was insufficient [72]. The awareness provided by the NGOs’ posts and the engagement of the public on the vital role of these medical professionals in preventing and eliminating GBV could lead to the health care sector and other types of leadership organizations finally taking ownership of their role in reducing GBV.

A current social issue represented as a subtheme under theme 4, GBV as a human rights violation, was the violence experienced by vulnerable groups in Mexico, including migrant girls, adolescents, and women. Since 2018, caravans of migrants from Central and South America have traveled to Mexico in order to reach the United States, with girls, adolescents, and women being particularly vulnerable to different forms of GBV through their travels in Mexico [73,74]. According to the literature, migrants are more likely to be the victims of human trafficking [73], another form of GBV also present in highly liked posts for the 3 campaign years. However, the posts analyzed did not provide resources that could help prevent human trafficking, as these posts only had data on the prevalence of this GBV. Specific resources, such as phone numbers, may be more instrumental, particularly in states such as Chiapas, where migrants tend to stay longer and where it has been shown that GBV is more prevalent for this group [73]. A vulnerable group not mentioned in the highly liked posts that were analyzed was of Indigenous girls, adolescents, and women, even though they have consistently experienced GBV, structural violence, and institutional violence [75,76]. It is unknown if posts with fewer likes and thus not included in these analyses specifically mentioned this vulnerable group in posts by NGOs during the 3 campaign years. Future research on this topic is suggested to not only better understand the public’s interest or awareness but also to identify different vulnerable groups that are not represented or addressed in nation-based campaigns or are not receiving broader support through recognition (likes).

Our results found that most of the messages on these posts exclusively mentioned women and younger girls, while only a few included adolescents. However, in Mexico, 60% of the adolescents aged between 15 and 17 years have experienced some type of GBV, and 40% have experienced sexual violence, while 80% of the minors aged <18 years who have disappeared in this country were adolescent girls aged between 12 and 17 years [4,77]. In addition, different studies have found a high prevalence of GBV in Mexican adolescent girls. For example, a study in the northern city of Tijuana found that most adolescent girls have experienced some form of intimate partner violence, more specifically, emotional, sexual, or physical abuse [78]. It is imperative for Mexico-based NGOs involved in future campaigns for 16 Days of Activism against GBV to be more inclusive of adolescent girls in their posts and have specific messages toward this age group, as they are vulnerable and in need of information to combat GBV, and to take account of the influence of social media toward adolescents. It is also important to state that the messages from most highly liked posts followed a collective voice, as these were written from a collective point of view in order to express ideas about GBV and activism against GBV through group unity, shared responsibility, and changes as a group. This characteristic of the highly liked posts from Mexican NGOs is in line with existing literature, which has previously identified that Latin American feminist movements are distinguished by a collective voice compared to non-Latin American campaigns that have been framed through an individualized lens, such as the #MeToo movement [79].

Research using social media data to learn more about hashtag activism and hashtag feminism has grown in the last decade. Particularly, published literature on X data to analyze activism against GBV in the world has focused on hashtags in English [38,80], and most of it has been related to sexual violence [22,39,40,81]. Previous examples of hashtag feminism in Mexico include the #MeToo movement in 2017; #SiMeMatan (#IfTheyKillMe), used by women in response to the media and authority’s secondary victimization of femicide victims; and #MiPrimerAcoso (#MyFirstAssault) in 2016, related to childhood sexual abuse, to name a few [82]. Some of these hashtags have been previously analyzed; however, to the best of our knowledge, we believe this research is one of the first studies to focus on Mexico-based NGOs, different campaign years, and more than 1 hashtag. This study provides important information on the types of messages that Mexico-based NGOs focused on in the prevention and elimination of GBV as well as what types of messages are potentially of more interest to the public. Future studies could further expand the limited knowledge on hashtag feminism against GBV in Mexico. For example, analyzing the demographics of the X users who liked these posts would provide context for understanding the population that follows this campaign. Analyzing other campaigns on GBV as well as the messages published and liked on other popular social media platforms in Mexico, such as Facebook, TikTok, and Instagram, is worth pursuing in future studies to further explore and better understand topics related to the prevention and elimination of GBV. Moreover, future research that quantifies post volume and interactive users through in-depth social network and semantic network analysis could provide more information on our findings by further exploring the level of user engagement and the evolution of discussions on GBV and activism over time. This tone of voice emerged across the 5 themes.

Finally, due to the inductive nature of this research, our study did not follow a theoretical framework. Nonetheless, our results lay the groundwork for future research that could benefit from the use of theoretical frameworks, such as the intersectionality framework [83], to further understand the intersecting identities of the public engaging in GBV campaigns and the feminist theory [84] to further study one of the main themes for this research, GBV as a violation of human rights, including gender inequities.

### Strengths and Limitations

As noted earlier, to the best of our knowledge, this study is the first of its kind to conduct a comprehensive thematic content analysis using X data to examine GBV activism across Mexico at a national level. It delivers a groundbreaking perspective on the powerful impact of hashtag activism and feminist movements, offering an unprecedented understanding of social activism in the country. The research brings valuable insights into the topics related to GBV and the messages from this activist campaign that the public engages with the most. This high-level synthesis provides an overview of priorities and topics that can help NGOs and other types of organizations be more strategic in their social media campaigns and messages to combat GBV. For example, we note a gap in the provision of specific resources for those facing GBV; this may be a key area for future campaigns to build on. Despite these strengths, we recognize the inherent limitations in working with X data. First, the text content of posts is limited to up to 280 characters. This particular characteristic of X could hinder the amount of content that can be shared with the public. While some posts analyzed in this study included videos or infographics that could further expand the message shared by the NGO, most posts were messages of <280 characters with concise and specific yet limited information. First, we chose to use X data for analysis instead of another platform such as Facebook, as X offers a diverse dataset and has been one of the main channels used for hashtag feminism [39]. Second, while we collected and analyzed all the text present in the highly liked posts, we only analyzed the visual elements from the posts in the form of pictures and short videos (<1 min), losing the potential for more emerging themes to come from longer videos. Third, our study is limited to posts posted by NGOs that participated in the 16 Days of Activism against GBV campaigns. The results are not generalizable to other types of users (such as government agencies and celebrities) or to other hashtags related to the campaign that were not analyzed in this research. Fourth, this analysis was specific to NGOs based in Mexico and posts published in Spanish, so our results might not be generalizable to the campaign in other countries. Fifth, we focused our research on 4 hashtags (and their variations) specific to this campaign; therefore, it is possible that our research missed capturing other themes from other hashtags (such as #10D, #niunamenos [#notonemore], #niunaasesinadamás [#notonemoremurdered]) related to the 2020, 2021, and 2022 campaigns. Sixth, because of the limited number of posts (N=600) analyzed in this research, themes could have been missed; however, a sample size of 500 posts has been demonstrated to be sufficient to identify themes and understand the public’s engagement [52,85]. Finally, the posts chosen for this analysis were based on the number of likes at the time of data collection. The number of likes on a post changes through time; therefore, the messages from these posts that generated the most attention and engagement from the public at the time of the campaign could have changed by the time the data were collected and might not reflect the engagement of the public with the different topics related to GBV. However, this is unlikely, as research has found that most likes on a post are given within 24 hours after the post is posted on X [86].

### Conclusions

Our findings reveal that the highly liked posts for the 16 Days of Activism against GBV campaigns posted by Mexico-based NGOs in 2020, 2021, and 2022 are a reflection of the forms of violence and social issues that currently occur in the country. According to our results, informational posts about the types of GBV, the role of activism, and changing public discourse surrounding GBV are the types of messages that seem to engage the public the most. Our results could help inform and guide future GBV campaigns while also informing the NGOs of the lack of representation in their messages about GBV toward important vulnerable populations, such as adolescents and Indigenous groups. Nevertheless, further research related to hashtag feminism by Mexico-based NGOs on GBV, such as the demographics of X users who engage on these posts and the types of activism interactions among X users on other social media platforms, is vital to understand the population that NGOs reach and how the messages shared on these campaigns translate into activism online and offline.

## Abbreviations

GBV: gender-based violence
NGO: nongovernmental organization
